# Two-Photon Excited Fluorescence Lifetime Imaging of Tetracycline-Labeled Retinal Calcification

**DOI:** 10.3390/s23146626

**Published:** 2023-07-24

**Authors:** Kavita R. Hegde, Krishanu Ray, Henryk Szmacinski, Sharon Sorto, Adam C. Puche, Imre Lengyel, Richard B. Thompson

**Affiliations:** 1 Department of Natural Sciences, Coppin State University, Baltimore, MD 21216, USA; 2 Department of Biochemistry and Molecular Biology, University of Maryland School of Medicine, Baltimore, MD 21201, USA; 3 Institute of Human Virology, University of Maryland School of Medicine, Baltimore, MD 21201, USA; 4 Department of Neurobiology, University of Maryland School of Medicine, Baltimore, MD 21201, USA; 5 The Wellcome-Wolfson Institute for Experimental Medicine, School of Medicine, Dentistry and Biomedical Science, Queen’s University Belfast, Belfast BT9 7BL, UK

**Keywords:** age-related macular degeneration, retina, calcification, drusen, hydroxyapatite, whitlockite, fluorescence lifetime imaging, chlortetracycline, doxycycline, FLIO, FLIM

## Abstract

Deposition of calcium-containing minerals such as hydroxyapatite and whitlockite in the subretinal pigment epithelial (sub-RPE) space of the retina is linked to the development of and progression to the end-stage of age-related macular degeneration (AMD). AMD is the most common eye disease causing blindness amongst the elderly in developed countries; early diagnosis is desirable, particularly to begin treatment where available. Calcification in the sub-RPE space is also directly linked to other diseases such as Pseudoxanthoma elasticum (PXE). We found that these mineral deposits could be imaged by fluorescence using tetracycline antibiotics as specific stains. Binding of tetracyclines to the minerals was accompanied by increases in fluorescence intensity and fluorescence lifetime. The lifetimes for tetracyclines differed substantially from the known background lifetime of the existing natural retinal fluorophores, suggesting that calcification could be visualized by lifetime imaging. However, the excitation wavelengths used to excite these lifetime changes were generally shorter than those approved for retinal imaging. Here, we show that tetracycline-stained drusen in *post mortem* human retinas may be imaged by fluorescence lifetime contrast using multiphoton (infrared) excitation. For this pilot study, ten eyes from six anonymous deceased donors (3 female, 3 male, mean age 83.7 years, range 79–97 years) were obtained with informed consent from the Maryland State Anatomy Board with ethical oversight and approval by the Institutional Review Board.

## 1. Introduction

Age-related macular degeneration (AMD) is the most common cause of irreversible vision loss among elderly persons in developed countries, with more than ten million persons affected in the United States alone. While the less common form (choroidal neovascularization, CNV) of AMD can be treated, treatments are just beginning to be introduced for the more common form (geographic atrophy, GA) (reviewed in [1,2,3]). Both forms are associated with the formation of extracellular lipid- and protein-containing deposits beneath the retinal pigment epithelial layer of the retina. The best known of these deposits are called drusen, which range up to tens of microns in size and are the hallmark lesions of AMD [4]; other important deposits are termed basal linear or laminar deposits [5]. These deposits can be visualized in the living retina by ophthalmoscopy and optical coherence tomography (OCT). It is widely believed that the buildup of these deposits beyond a certain level interdicts the flux of oxygen, nutrients, to the metabolically active rods, cones, and neurons in the retina, leading to their atrophy and death in AMD. Due to the role of these deposits in the development and progression of AMD, understanding their etiology and potential contribution to AMD pathology is important for developing new diagnostic and therapeutic approaches. It has been demonstrated that sub-RPE deposits contain microscopic calcium phosphate minerals, particularly hydroxyapatite (HAP) and whitlockite (WHT) [6,7]. The minerals frequently form spherules of diameter circa one micron and are coated with proteins characteristic of drusen; we thus proposed that these mineral deposits nucleate the growth of drusen in the retina and may indicate the potential for disease [6]. Subsequently, we showed that larger calcified retinal deposits (termed nodules) are an important risk factor for developing advanced AMD (either geographic atrophy or choroidal neovascularization) within one year (odds ratio 6.4:1) [8].

These findings kindled interest in imaging these mineral deposits in situ to understand their origins and relationship to disease. The rarer large HAP nodules (tens of microns) provide good contrast for multimodal imaging; however, even with adaptive optics imaging techniques, the micron-sized spherules are too small to resolve by OCT or standard ophthalmoscopy. The most commonly used visualization methods for tissue calcification elsewhere in the body like the lungs, kidneys, or heart are radiography or ultrasound, but at present, their resolutions are much poorer than the micron levels needed to detect early calcification in the retina. Since fluorescence imaging of the retina in the living eye by ophthalmoscopy is well developed and can resolve micron-sized targets (e.g., fundus autofluorescence studies, fluorescein angiography, etc.), we considered means to image the calcification using fluorescence methods.

Neither HAP nor WHT has appreciable fluorescence on its own but can be labeled by stains that selectively bind calcium phosphate minerals and fluoresce. There are selective fluorescent stains for HAP (e.g., Bone-Tag and OsteoSense), some of which offer good sensitivity by emitting at near infrared wavelengths (up to and above 810 nm), where retinal tissue autofluorescence is usually minimal. Due to their excitation at NIR wavelengths, these dyes could be comfortable and safe for imaging in patients’ eyes and reduce scattering for improved image quality [9,10]. However, these stains are less attractive for in vivo studies since their pharmacokinetics, toxicity, and ADME (absorption, distribution, metabolism, and excretion) properties are largely unknown, particularly in primates. Moreover, the heptamethine cyanine fluorescent moieties of these infrared dyes are noted as photosensitizers [11] and are likely to require intravenous injections in their current forms, potentially compromising safety.

Some tetracycline antibiotics are known to bind to HAP with an accompanying fluorescence intensity increase. Tetracycline antibiotics are widely considered safe and have well-known pharmacokinetics, toxicity, and ADME profiles; moreover, nearly all can be administered orally [12,13,14]. We have shown that binding of certain tetracyclines to HAP was accompanied by a significant increase in apparent quantum yield and fluorescence lifetime [15]. Using fluorescence lifetime measurements, we were able to distinguish ex vivo drusen in images of retinas stained with chlortetracycline (Aureomycin) from the background fluorescence of the Bruch’s membrane in *post mortem* retinal tissues (chlortetracycline bound to HAP = 1.6 ns, Bruch’s membrane background = 0.4 ns; reviewed in [16]) using fluorescence lifetime imaging microscopy (FLIM) [15,17].

FLIM is a fluorescence microscopy method where the contrast in the images arises from differences in the fluorescence lifetime of the emitting species rather than the intensity or color (wavelength) of the fluorescence, as in conventional fluorescence microscopy. We subsequently showed that chlortetracycline could label drusen in the retina via systemic perfusion, wherein the compound is introduced into the bloodstream of a cadaver and allowed to circulate using an external pump [18]. These results suggested that calcification in the retina might be labeled by orally administered tetracycline antibiotics, whence they would enter the bloodstream as usual, and be imaged by FLIM. The pioneering development of fluorescence lifetime imaging ophthalmoscopy (FLIO) by Schweitzer, Zinkernagel, and their colleagues and its demonstration in living humans (reviewed in [16]) demonstrated that lifetime imaging is feasible in situ in the living human retina. Therefore, the prospect emerged of orally administered tetracycline and the use of FLIO perhaps being used to predict early drusen formation through monitoring calcification by imaging tetracycline-stained HAP in the retina, allowing the significantly earlier identification of the initial phases of age-related macular degeneration.

By comparison with chlortetracycline, doxycycline (Doxy, Sigma, St. Louis, MO, USA; CAS [17086-28-1]) offers even better lifetime resolution when bound to HAP (3.8 ns compared to the 0.4 ns of tissue background). However, there is a significant drawback: the maximum of its excitation is at about 390 nm. This is problematic both because of the overt safety issue of illuminating the eye with ultraviolet light, but also because the shorter wavelength excitation very likely will produce greatly increased autofluorescence background, especially in the aqueous and vitreous humors, lens, and cornea, as it does in other tissues. We addressed these issues using a technique relatively new to ophthalmology, multiphoton fluorescence excitation.

Multiphoton absorption was predicted theoretically by Göppert-Mayer [19] and first applied to fluorescence microscopy by Webb and colleagues [20]. Under conditions of very high peak power (typically realized in ultrashort mode-locked laser pulses in the pico- to femto-second ranges), a molecule can simultaneously absorb two (or more) longer wavelength photons as if they were a single photon of twice the energy and half the wavelength and be raised to an excited state. For instance, a molecule like fluorescein that does not absorb light at all at 800 nm can nonetheless be excited by light at that wavelength if it is in the form of extremely brief pulses of light typically lasting trillionths of a second or less, such that the peak power delivered is very high. If fluorescent, it will ordinarily emit as if it had absorbed a single photon of shorter wavelength/higher energy in the usual way, exhibiting the same emission spectrum as if it had been excited with a photon it can readily absorb at 400 nm. Importantly, the propensity of a fluorophore to be excited by multiple, longer wavelength photons is not related in any simple way to its absorption spectrum but can be predicted with difficulty from the structure. This property is called the two- (or multi-) photon cross-section. The peak values of the cross-section, expressed in units of Göppert-Mayer (G-M), can vary from above 10,000 G-M for some squaraine-rotaxanes [21] down to approximately 1 G-M for NADH [22]. While the pioneering work of Atmeh, et al. [23] showed that tetracycline itself at least exhibits multiphoton excited fluorescence, it was unclear whether Chlortetracycline or Doxycycline would as well when bound to HAP, and what lifetimes they would exhibit. Thus, we performed a feasibility study testing staining of retinas removed from cadavers with Chlortetracycline and Doxycycline, either by staining flatmounted retinas or staining intact retinas in cadavers by infusion as we previously described [18] and imaging them by FLIM with 2 photon excitation. Multiphoton excitation of fluorescence and its application to microscopy are reviewed in [20,24].

## 2. Materials and Methods

For this pilot study, ten eyes from six anonymous deceased donors (3 female, 3 male, mean age 83.7 years, range 79–97 years) were obtained with informed consent from the Maryland State Anatomy Board with ethical oversight and approval by the Institutional Review Board. Cadavers were typically perfused through the carotid artery 6–16 h *post mortem* with 500 mg/L chlortetracycline (0.96 mM) or 250 mg/L doxycycline (0.56 mM) in PBS for one hour, followed by a one-hour washout with PBS only. These concentrations were chosen to approximate human doses administered to treat infection. We note that this procedure does not exactly simulate infusion in a living human; nevertheless, it is an appropriate simulation of staining the sub-RPE deposits of the retina by tetracyclines in the circulation. The eyes were then enucleated, and the front of the eye was removed by incision following the limbus to allow the neural retina and RPE to be removed, and the retinas flatmounted for FLIM, all essentially as previously described [18]. The specimens were imaged with an ISS Q2 (Urbana, IL, USA) fluorescence lifetime imaging microscope through 20 × 0.7 NA or 60 × 1.2 NA objectives with a Calmar modelocked fiber optic laser emitting 780 nm excitation pulses of 90 fs duration at 50 MHz repetition rate with average power up to 12 mW and emission photons detected by solid-state detectors. The arrival times of emission photons were collected and stored as a histogram using time-correlated single photon counting electronics essentially as described in [24].

Time-resolved data were analyzed and depicted using VistaVision software by ISS: the data are analyzed by fitting the obtained time-dependent decay in the fluorescence intensity to an assumed mixture of two or more fluorescence lifetime components, to extract their lifetime values and relative proportions in the mixture. Time-correlated single photon counting (TCSPC) histograms for individual pixels are depicted as usual as the logarithm of the time-resolved intensity as a function of time and were mostly fit by a standard method to a two-exponential decay model using the Marquardt algorithm, by minimizing the sum of the squares of differences between the measured and calculated values of time-dependent intensity and calculating a reduced χ2. Two components were usually chosen because it is clear from the raw data there is more than one component (the decay lines would be straight on the logarithmic plot if there were a single fluorescing component), and while it is likely there are multiple fluorescent lifetimes present, we have no ab initio reason to presume there are exactly 3 or 4 lifetimes present. For some images, single component fits providing a single lifetime were used rather than the average of two components for computational tractability. In the time-resolved fluorescence decays depicted in Figures 2 and 3, the blue dots are the data from the individual time intervals and the red line indicates the decay curve calculated from the best fit using the pre-exponential factors α_1_ and α_2_, and lifetimes τ_1_ and τ_2_, as described in the text. Directly underneath, the decay curves in Figures 2 and 3 are plotted for each time point, in units of standard deviations, the difference between the actual measured intensity and the calculated value from the fit at that time point. If a fit is good, the differences (called residuals) are mostly small (90% < 2 standard deviations) and randomly distributed around zero, and the derived χ2 is near to 1.0; in simple terms, the red line passes through the blue dots. If a fit is poor, the differences are larger, vary systematically from zero, and the χ2 is larger. An excellent introduction and didactic presentation of the collection and analysis of time-resolved fluorescence data and FLIM may be found in [24].

## 3. Results

The broad goals of this pilot study on multiphoton excitation imaging of tetracycline-stained retinas were to test the feasibility of the procedure to be used in vitro, and to assess effect sizes. Ten eyes from six donors (3 female, 3 male, mean age 83.7 years, range 79 to 97 years) were employed in the study.

**Figure 1:**
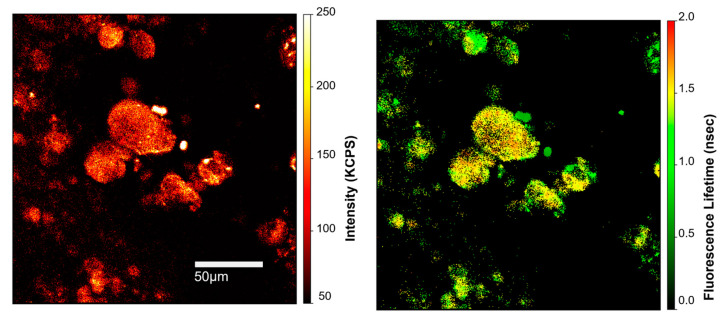
Two-photon-excited fluorescence intensity micrograph (**left panel**) with color-coded intensities (arbitrary units) on the right side of the panel, and fluorescence lifetime micrograph of the same field (**right panel**) fit to a single component with time indicated by false colors with scale in nanoseconds on the right. Experiments were carried out on an infusion-stained, flat-mounted retina, following the removal of the RPE and neurosensory retina (97-year-old female donor; cause of death: cardiovascular disease). Sample was labelled with Cl-Tet. Approximate size of the field: 250 × 250 μm^2^; further details can be found in the Methods.

Figure 1 depicts fluorescence intensity and fluorescence lifetime micrographs of a representative flatmounted retina from a 97-year-old Black female donor with the neural retina and RPE removed, exposing the Bruch’s membrane–choroid complex stained with Cl-Tet. There are several drusen in the field of view and they stand out in both the intensity and lifetime images. Whereas the intensities of the drusen in Figure 1(left panel) are two- to three-fold larger than the background, the lifetimes of the drusen on Figure 1(right panel) are greater than five-fold longer than the lifetime of the background. This suggests that the fluorescence lifetime approach offers additional contrast (and potentially better sensitivity) for detecting calcification in the retina.

**Figure 2:**
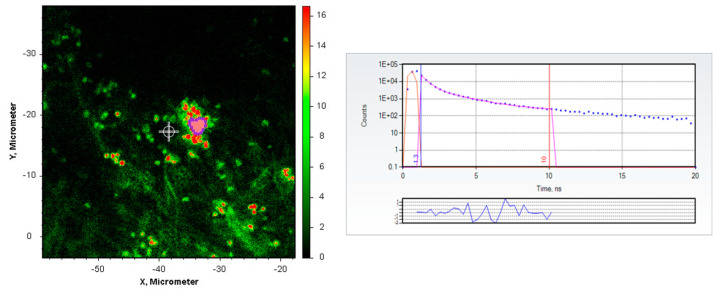
Fluorescence intensity micrograph (**left panel**) of doxycycline-stained drusen in the retina of a 79-year-old white male donor (cause of death: chronic myelocytic anemia); the intensities are false colored according to the scale (arbitrary units) on the right of the image. The (**right panel**) shows the time-resolved fluorescence decay of the aggregated pixels in the indicated region of interest (pink); The purple dots indicate the individual time-resolved fluorescence intensity data points, the red line through the dots indicates the best fit to the data, the orange curve before two nanoseconds indicates the instrument response function, and the vertical lines at 1.3 and 10 nanoseconds indicate the beginning and end, respectively, of the data included in the fit. The solid purple line in the lower part of the right panel depicts the “residuals”: the differences between the actual measured data points and the values calculated for that time point by the best fit parameters.

We also labeled retinas by transarterial infusion *post mortem* with doxycycline solution, using a similar protocol but less than 250 mg antibiotic/L in the buffer, since even the doses of doxycycline administered prophylactically to individuals exposed to Lyme disease are smaller than for Cl-Tet. After perfusion with doxycycline, removal of the neurosensory retina and RPE, and flatmounting, robust staining of drusen (bright shapes) was observed with two-photon excitation and fluorescence lifetime imaging (Figure 2).

Figure 2 depicts a fluorescence intensity micrograph (further details in Methods) of several small drusen in the retina of a 79-year-old white male donor (cause of death: chronic myelocytic leukaemia) stained with Doxycycline by infusion and imaged with two-photon excitation at 780 nm. The right panel in Figure 2 depicts the best two-component fit to the decay in the pink highlighted region of interest centered at X = −34 μm, Y = −18 μm, yielding τ_1_ = 0.430 ns, α_1_ = 0.88, τ_2_ = 2.111 ns, α_2_ = 0.12, χ2 = 0.88. The bright spots in the small drusen were reminiscent of the micron-sized HAP spherules we had previously observed in drusen [6]. We therefore decided to image them further with the FLIM at higher magnification (60×).

**Figure 3:**
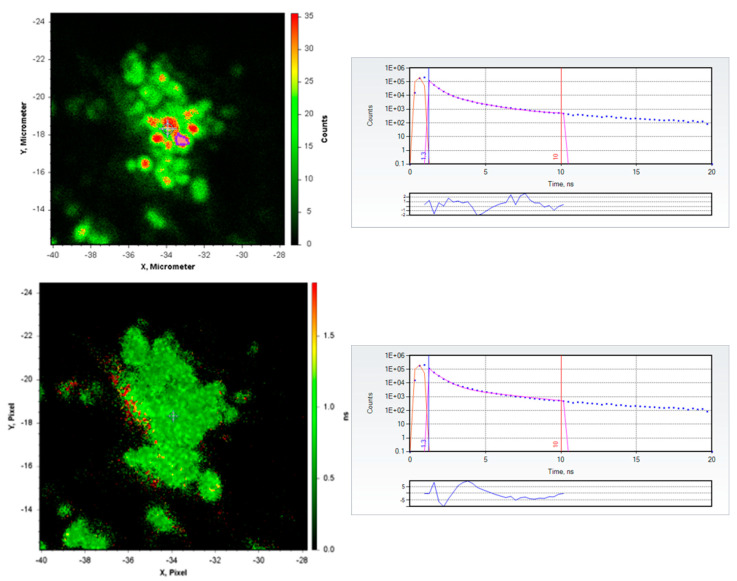
Close-ups of the druse in Figure 2 at higher (60×) magnification ((**upper left panel**): fluorescence intensity in false color with scale on right side in arbitrary units; (**lower left panel**): single component fluorescence lifetime in false color with scale on the right in nanoseconds) and best two component fits to the pink region of interest at X = −33 μm, Y = −17 μm in the upper left panel, with both lifetimes floating (**upper right panel**), and with one lifetime fixed to 3.7 ns (**lower right panel**). The upper and lower right panels depict the decays using the same conventions as described in the Figure 2 legend.

The images in Figure 3 depict a close-up with a 60× objective of the prominent druse near the center of Figure 2. The small round blobs clustered in the center of the image are reminiscent in shape and size of the calcium phosphate spherules we had previously observed [6]; we note they are distinctly smaller than and not polygonal like RPE cells. We fit the time-resolved fluorescence decay of the pink region of interest highlighted in the upper left panel of Figure 3 at X = −33 μm, Y = −17 μm, to two fluorescing components, with the data compared to the best fit in the upper right panel: the derived values were τ_1_ = 0.417 ns, α_1_ = 0.95, τ_2_ = 1.737 ns, α_2_ = 0.05, χ2 = 2.06. We sought to determine if the fit would be significantly worse if the value of the long component were fixed at 3.7 ns, the lifetime value we had previously determined for doxycycline bound to hydroxyapatite. The returned values from this fit were τ_1_ = 0.562 ns, α_1_ = 0.97, τ_2_ = 3.7 (Fixed) ns, α_2_ = 0.03, χ2 = 29. As is apparent from the higher χ2 and the larger and systematically varying residuals in the lower right plot in Figure 3, the fit is not as good. There are several possible explanations why we did not observe a 3.7 ns long component: it may be that the infusion staining procedure we used for doxycycline is not as effective as that for chlortetracycline, or that the doxycycline is preferentially adhering to another component of the drusen whence it does not exhibit the 3.7 ns component. The latter is likely given the known propensity of tetracyclines to adhere to proteins in the circulation and elsewhere [13,14]. Further experimentation will be necessary to resolve this question and is underway. If we fit the entire image in Figure 3 to a single exponential, we obtain the image in the lower left of Figure 3, where the small regions in the druse exhibiting a range of fluorescence intensity are remarkably uniform in their apparent fluorescence lifetime properties. However, with doxycycline staining as well, it is evident that the fluorescence intensities and lifetimes of the stained drusen are several-fold greater than the background fluorescence emission, suggesting they will be potentially useful for early imaging of drusen.

## 4. Discussion

It is worth asking: why go to the trouble and expense of using multiphoton excitation to image fluorescence in the retina instead of one photon excitation, in this case, of tetracycline-stained subretinal deposits? The principal reasons are safety and reduced background. Near-infrared (NIR) excitation in the form of picosecond/femtosecond pulses in the neighborhood of 800 nm is invisible (as it is for optical coherence tomography (OCT) and NIR reflectance), and this reduces both risk and patient discomfort. It is noted that NIR psec/fsec laser pulses, but at much higher powers, are now widely used for several types of ophthalmic surgery, including corneal, cataract, and refractive procedures (reviewed in [25]). The successful development of fluorescence lifetime imaging ophthalmoscopy (FLIO) suggested in this case that better contrast with the background might be obtained with lifetime images than intensity images in view of the large ratio of fluorescence lifetimes of the HAP-bound chlortetracycline (1.6 ns) and doxycycline (3.7 ns) compared to the observed autofluorescence lifetime of the retina (0.2–0.4 ns). A third advantage lies in the intrinsic confocality of the two-photon excitation approach: with one photon excitation, in a confocal ophthalmoscope, the pinhole(s) reduce off-axis stray fluorescence, improving contrast and resolution. However, background fluorescence along the optical axis arising from the cornea, lens, and vitreous is captured and degrades the image. By comparison, with two-photon excitation, the fluorescence intensity is proportion to the square of the peak excitation intensity, which is highest at the focal plane even at modest numerical apertures, and the on-axis excitation outside the (narrow) focal plane and thus background is thereby reduced.

It was notable that while previously [15], and in Figure 1, we showed that a substantial portion of the druse (perhaps in addition to the mineral) exhibited a fluorescence lifetime comparable to that of HAP stained with Cl-Tet, the lifetime (or long lifetime component) of doxycycline-stained drusen did not exhibit a significant long lifetime component of the value we expected. There are multiple potential explanations for this. It may be that the bulk of the doxycycline (like at least a portion of the Cl-Tet, we believe) adheres to lipid and protein species in the druse as well as HAP and exhibits a less prolonged lifetime in that instance. In addition, it may be that under our conditions, doxycycline is a less effective stain than Cl-Tet, and the conditions must be modified in living animals or humans to stain the minerals better; we note that we have demonstrated that aged macaques which develop drusen in their retinas exhibit hydroxyapatite staining comparable to that in humans (manuscript in preparation). Finally, there are many tetracycline analogs known to the art, some of which may also exhibit favorable fluorescence properties for such staining applications.

The innovative experiments by the Hunter [26] and Palczewski groups [27] demonstrate the feasibility and safety of two-photon excitation of fluorescence in the living retinas of animal models and, more recently, in living humans. One manufacturer has shown the adaptation of a short-pulse laser to a commercial scanning laser ophthalmoscope [28] for multiphoton excitation; the principal change required (in addition to the laser and filters) was the insertion of optics to correct for the effects of wavelength dispersion on the pulse duration.

We also note that there are different ways of depicting lifetime images or, more particularly, generating an image where the contrast is based on fluorescence lifetime properties. For practical use, recovering the actual values of the lifetimes of individual pixels may not be important: rather, we are interested in identifying pixels in the image where the stain is adhering to calcification in the drusen, which is essentially a bimodal, black/white mapping. For this purpose, use of the phasor plot reintroduced by Redford and Clegg [29] that enables pixels exhibiting particular lifetime properties to be highlighted is well suited [15]. For lifetime data acquired in the frequency domain, the use of classic phase-sensitive detection at a suitable modulation frequency [30] can also be used in an imaging format to discriminate between pixels on the basis of their lifetime properties [17].

As a pilot study, this work has clear limitations but points the way to future experiments and potential clinical utility. The small subject cohort makes it infeasible to arrive at statistically useful conclusions, and the *post mortem* specimens are perforce not living tissue, but our goal here was to assess feasibility and effect sizes with available resources and instrumentation. Based on the FLIO literature, it is likely that the substantial lifetime contrast of tetracycline-stained calcification we observed by FLIM in flatmounted tissue here could be imaged in the retina in vivo by FLIO using two-photon excitation. We are beginning a FLIO study in aged non-human primates that exhibit drusen to assess the efficacy and safety of our approach. While we and others as described in the Introduction have developed evidence suggesting that imaging properties of HAP deposits may be useful for early prediction of AMD, this remains to be shown definitively and will require studies in humans. Nevertheless, it is not much of a stretch to imagine that a rapid clinical retinal imaging test for HAP following oral administration of a legacy antibiotic that predicted the onset and progression of AMD in time to begin treatment before vision loss is a desirable goal.

Finally, we are grateful to one of the reviewers for suggesting that some of our readers might find it useful to briefly compare the techniques and instruments of FLIM (fluorescence lifetime imaging microscopy) and FLIO (fluorescence lifetime imaging ophthalmoscopy), since the former has become widely available and the latter is just being introduced. The two techniques are fundamentally similar, in that they construct an image typically collected by scanning the target (either a microscope specimen for FLIM, or the retina in vivo for FLIO) with an exciting, short pulse laser beam and timing the arrival of emitted fluorescence photons from each pixel, where the contrast in the image arises from differences in fluorescence lifetime of the individual pixels. At first glance, the FLIM and FLIO instruments look and operate much like their familiar steady state counterparts, but the data (i.e., Figure 1(right panel) and Figure 3(lower left panel)) provide fundamentally different information than the intensity and color seen in typical fluorescence images. Optically, FLIM and FLIO instruments are not too different from typical scanning epifluorescence microscopes (such as confocal or multiphoton microscopes) or scanning laser ophthalmoscopes, respectively. Of course, they differ in that the microscope is used on small, isolated, sometimes nonliving specimens such as cells or tissues (as in our study), whereas the FLIO is designed for imaging the retina through the intact lens, cornea, and the rest of the eye’s optical path in a living subject. Obviously, this confers additional requirements on the FLIO’s peak excitation intensity, wavelength, and other features for the safety and comfort of the human subject. The characteristics of several examples of FLIO lifetime retinal images (and how they differ from other retinal imaging methods such as OCT, FAF, fluorescence angiography) can be seen in Reference [16].

## Data Availability

Inasmuch as the data were obtained partly with NIH funding, data will be archived and made available to other research pursuant to their regulations.
